# Deep Penetration Microscopic Imaging with Non-Diffracting Airy Beams

**DOI:** 10.3390/membranes11060391

**Published:** 2021-05-26

**Authors:** Yong Guo, Yangrui Huang, Jin Li, Luwei Wang, Zhigang Yang, Jinyuan Liu, Xiao Peng, Wei Yan, Junle Qu

**Affiliations:** College of Physics and Optoeletronic Engineering, Key Laboratory of Optoelectronic Devices and Systems of Ministry of Education and Guangdong Province, Shenzhen University, Shenzhen 518060, China; 1800284004@szu.edu.cn (Y.G.); 1900453023@szu.edu.cn (Y.H.); 1910454084@email.szu.edu.cn (J.L.); wangloell@szu.edu.cn (L.W.); zhgyang@szu.edu.cn (Z.Y.); ljy@szu.edu.cn (J.L.); pengxiao_px@szu.edu.cn (X.P.); jlqu@szu.edu.cn (J.Q.)

**Keywords:** deep penetration, microscopic imaging, non-diffracting Airy beams, dynamic volumetric imaging, acquisition speed

## Abstract

We report a deep penetration microscopic imaging method with a non-diffracting Airy beam. The direct mapping of volume imaging in free space shows that the axial imaging range of the Airy beam is approximately 4 times that of the traditional Gaussian beam along the axial direction while maintaining a narrow lateral width. Benefiting from its non-diffracting property, the microscopic imaging with Airy beam illumination can acquire image structures through turbid medium and capture a volumetric image in a single frame. We demonstrate the penetration ability of the Airy microscopic imaging through a strongly scattering environment with 633 nm and 780 nm lasers. The performances of the volumetric imaging method were evaluated using HeLa cells and isolated mouse kidney tissue. The thick sample was scanned layer by layer in the Gaussian mode, however, in the Airy mode, the three-dimensional (3D) structure information was projected onto a two-dimensional (2D) image, which vastly increased the volume imaging speed. To show the characteristics of the Airy microscope, we performed dynamic volumetric imaging on the isolated mouse kidney tissue with two-photon.

## 1. Introduction

Laser scanning microscopy (LSM) is an optical imaging technology that was developed in the mid-1980s. Due to its high resolution and good tomography capability, it has become an essential tool for biomedical experimental research. However, the limited penetration depth of the optical microscope makes it difficult to observe thick tissues. The main reason for this limitation is the strong optical scattering of the biological organelles. To overcome this challenge, researchers have proposed many methods, including Airy beams. A distinctive property of the Airy beam is the ability to fully recover after encountering an obstacle and to maintain its shape over a long propagation distance. It is natural to think of using Airy beams to increase the penetration depth of LSM. The confocal microscope as a typical LSM, a highly focused laser is used to scan the sample point by point three-dimensionally (3D), and a pinhole is used to block the fluorescence signal out of the focal plane and only allows for the detection of fluorescence signal from the focal plane to achieve 3D tomographic imaging [1,2,3]. However, temporal resolution is greatly limited by this scanning imaging mode, which seriously reduces the volume frame rate. A slow frame rate can also have the side effect of motion blur when imaging live samples, therefore it is not suitable for observing live biological samples for a long time with phototoxicity and photobleaching. For 3D optical microscopy, the important characteristics not only contain high spatial and temporal resolution but also have good imaging quality and depth. The aberrations caused by the microscopic system or the sample will reduce the image contrast and signal-to-noise ratio (SNR), which affects the imaging quality. Conventional adaptive optics technology can be used to correct the aberrations caused by the sample and the system [4,5,6,7]. Although the improved image quality is obvious after correction, it remains limited. Additionally, due to the strong scattering of biological organelles and the absorption of biological tissues, the penetration depth of optical microscope imaging is limited [8], so it is a great challenge to see the structure of thick biological samples and understand their internal working principles. Many techniques have been developed to image thick organisms using different principles, such as light-sheet microscopy, two-photon microscopy, and multi-photon microscopy [9,10,11]. As the absorption and scattering of near-infrared light by biological tissues is weaker than that of visible light, two-photon or multi-photon fluorescence microscopy using near-infrared ultra-short pulse laser as the light source can significantly improve the imaging depth, and a low-power continuous infrared laser can also reduce the light damage, but increasing the fluorescence wavelength will reduce the spatial resolution [12]. Light-sheet microscopy can acquire tomographic images at different depths and realize 3D information reconstruction of samples. It has the advantages of 3D imaging, high contrast, and low light damage, but the spatial resolution and the field of view are mutually restricted [13]. Some researchers have generated a new type of light sheet with a non-diffracting Bessel beam to solve the problem of mutual restriction between the observation field and the axial resolution of the light sheet fluorescence microscopy system. For example, Planchon and coworkers generated uniform intensity Bessel light sheets using the zero-order Bessel beams that maintained their shape over long propagation distance [14], however, the defocusing background noise produced by the concentric ringside lobe of the Bessel beam reduces the imaging contrast and system axial resolution and increases the photobleaching and phototoxic effects. Fahrbach and coworkers combined the confocal technology to solve the problems of low contrast and axial resolution in Bessel light-sheet fluorescence microscopy imaging [15]. But hundreds of low-contrast images must be acquired to reconstruct a high-contrast three-dimensional image by this method, and the imaging speed of the system decreases by 2 orders of magnitude. To solve the problem of 3D imaging speed, Airy beams volumetric imaging has attracted attention. Airy and Bessel beams are the two solutions to the Helmholtz wave equation [16,17]. They have the characteristics of being non-diffracting and maintaining their shape over long propagation distances. However, another remarkable property of Airy beams is that they can completely recover after encountering a partial obstacle [18]. Furthermore, Airy beams have self-healing and self-acceleration properties, which are two of the more attractive ones [19,20]. For example, Tan and coworkers demonstrated volumetric imaging using two-photon Airy beams [20]. The special features of Airy beams have resulted in many other applications. In 3D super-resolution microscopy, the self-acceleration-induced bending trajectory has been applied to the axial localization of molecules [21,22]. The self-healing and non-diffracting properties of Airy beams have been used to extend the field of view in light-sheet microscopy, for example, Vettenburg and coworkers reported that the accelerating Airy beam innately yields high contrast and resolution up to a tenfold larger field-of-view [9]. The microscope of Airy beams can also image through turbulent medium and a highly scattering environment [23,24]. Although the two-photon Airy beams volumetric imaging has been reported by Ref [20], there are few reports about the studies of near-infrared light penetration ability in scattering media and two-photon dynamic volumetric imaging in biological tissues with Airy beams. Therefore, in this paper, we developed a non-diffracting Airy beam microscopic imaging system with 633 nm and 780 nm lasers. Using this system, we not only investigate the penetration ability of the non-diffracting Airy beam microscopic imaging through a scattering environment but also study the volumetric image performance of the non-diffracting Airy beam using HeLa cells and the isolated mouse kidney tissue, then using the two-photon volumetric imaging of Airy beam to dynamic monitor the changing of the isolated mouse kidney tissue. The volumetric sample is scanned layer by layer in the Gaussian mode, while the 3D structure information is projected onto a single 2D image in the Airy mode, which increases the acquisition speed significantly and avoids the side effect of motion blur in in vivo imaging.

## 2. System Set-Up

**Figure 1:**
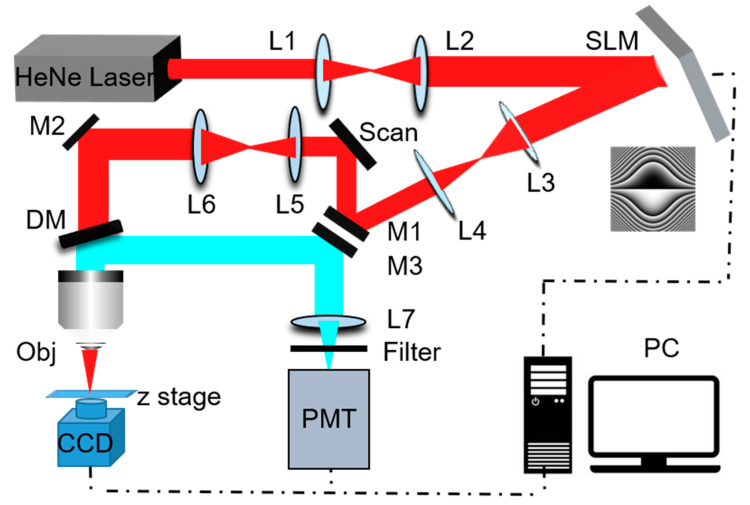
Schematic of the experimental setup. L1–L7: lenses; SLM: spatial light modulator; DM: dichroic mirror; M1–M3: mirrors; Scan: X,Y linear scan; CCD: charge-coupled device camera; PMT: photomultiplier tube; Obj: Olympus fluorite objective; Z stage: motorized precision translation stage. The two-photon Airy beams imaging setup of 780 nm laser is not listed.

Figure 1 is the schematic of the experimental setup, in which a HeNe laser (CW laser with power 5 mW) emitting 633 nm light is expanded by a pair of lenses (L1: 200 mm, L2: 300 mm). The collimated beam is modulated by the SLM(Spatial light modulator, Holoeye pluto2) with the Airy mask, which converts the Gaussian beams into the Airy beams. The SLM is conjugated with a pair of X–Y galvanometric scanning mirrors (California Institute of Technology, 6210H) through a 4f system (L3:300 mm, L4:200 mm). The X–Y galvanometric scanning mirrors are used to scan the beam across the sample, and its operating frame rate (512*512) is up to 2 Hz. The galvanometric scanning mirror through a second 4f system (L5:125 mm, L6: 200 mm) is further conjugated to the rear aperture of a fluorite objective (Olympus, X10, NA = 0.3). Airy and Gaussian beams can be reconstructed by recording the light intensity in different z planes using a CCD which can be moved along the optical axis by a motorized precision translation stage (z stage). The fluorescence signal is collected by the same objective and separated from the major beam path by a dichroic mirror (Chroma, ZT640RDC), then, is focused into the PMT (H7422-40 Hamamatsu) by a lens (L7:125 mm). Between the PMT and the lens (L7) is a bandpass filter (Chroma ET690/50) which can remove any residual excitation light. The Gaussian and Airy modes can be achieved by switching the SLM on and off.

## 3. Results and Discussion

**Figure 2:**
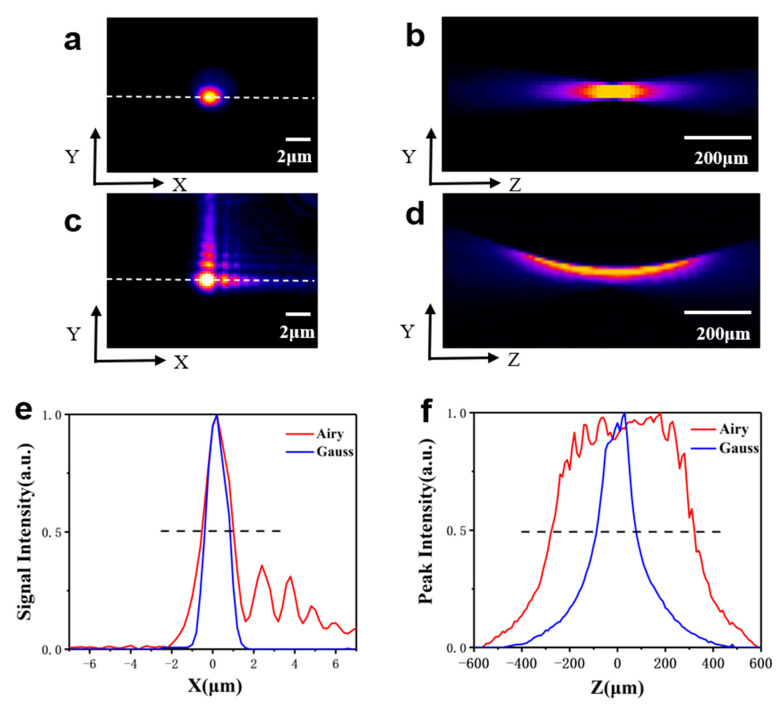
Images of Gaussian (top) and Airy (middle) spot shapes in free space with 633 nm laser. (**a**) and (**b**) are lateral and axial images in the Gaussian mode, respectively; (**c**) and (**d**) are lateral and axial images in the Airy mode, respectively; (**e**) Intensity profiles in lateral images marked with white dotted lines in (**a**) and (**c**); (**f**) Maximum signal intensity along the axial direction for (**b**) and (**d**). Red lines indicate the Airy beam, blue lines stand for the Gaussian beam. Both signal intensities of the Airy beam and Gaussian beam are normalized.

When using the non-diffracting Airy beam in our imaging system, we focus on several of their characteristics. First, the length of the focal beam in the axial direction, which determines the upper limit of the imaging penetration depth, should be as large as possible. Second, the beam waist should be as narrow as possible because it determines the lateral resolution. Therefore, it is very important to explore the free space characteristics of Gaussian and Airy beams. These beams are created through an objective (Olympus 10X NA = 0.3) and motorized stage (SUTTER MCP285), while the intensity distribution of light beams are captured by CCD (MER-040-60UM IMAVISION). The images of Gaussian and Airy beams focus spots in free space are shown in Figure 2. The lateral images of the Gaussian and Airy beams are shown in Figure 2a,c, respectively, and the intensity profiles marked with the dotted lines are shown in Figure 2e. The size of the main lobe of the Airy image (full width at half-maximum, FWHM: 1.4 μm) is approximately identical to that in Gaussian mode (FWHM: 1.3 μm). Furthermore, we map the axial images of the beam by superimposing the lateral images. The axial projections of the images are shown in Figure 2b,d for the Gaussian and Airy beams, respectively. In comparison, the Airy beam is much longer than the Gaussian beam along the axial direction. The maximum signal intensities of the two trajectories along the axial direction are shown in Figure 2f. The FWHM of axial is 165 μm for the Gaussian mode and 596 μm for the Airy mode. The FWHM of the axial for the Airy beam is approximately 3.6 times larger than that of the Gaussian beam, which indicates a significant broadening of the axial imaging range. Therefore, the optical penetration depth and volume imaging with Airy beams are worth exploring.

**Figure 3:**
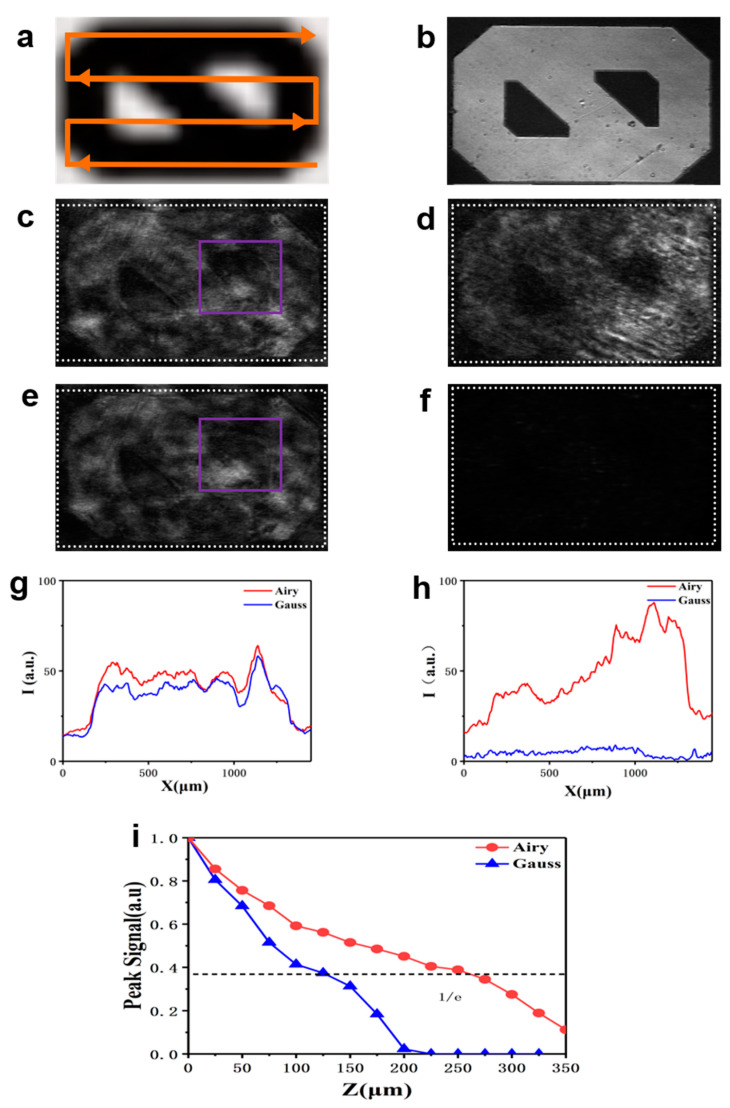
Relative intensity of the reflected light images from the target by line scanning with laser (633 nm) Airy and Gaussian beam. (**a**) Scanning method; (**b**) Image of the reflected light from the sample with no media; (**c**), (**e**) Images obtained with the Airy and Gaussian beams through a 150 μm thick water/milk mixture, respectively; (**d**), (**f**) Images obtained with the Airy and Gaussian beams through 300 μm thick water/milk mixture, respectively; (**g**) and (**h**) show the relative intensities of the reflected beam from (**c**) and (**e**) as well as (**d**) and (**f**) (the intensities projection along the long side in white dotted box), respectively: red is Airy and blue is Gauss; (**i**) Peak signals of reflected light at different thickness water/milk mixture for the Airy beam (red circles) and the Gaussian beam (blue triangles) microscopic imaging. Both signal intensities of the Airy beam and Gaussian beam are normalized.

Next, we conducted experiments through turbid media with the single-photon (633 nm) Airy and Gaussian beam to assess the penetrating ability of light spots. Our turbid media for imaging are glass chambers, approximately 150 μm and 300 μm thick, filled with a water/milk mixture (6% fat milk). At the end of our samples is a micrometer target (Micrometer, R1L3S1P Thorlabs) which is imaged through the turbid media. A segment of the target and scanning mode is shown in Figure 3a. We imaged using the reflected light of the sample at each scan position (Figure 3b). Figure 3c,e show images under the Airy beam and Gaussian beam through a 150 μm thick water/milk mixture, respectively. Figure 3d,f show images under the Airy beam and Gaussian beam through 300 μm thick water/milk mixture, respectively. We record the signal intensity image by line scanning for imaging. Figure 3g shows the relative intensity of the beam reflected through a 150 μm thick water/milk mixture over the target: red is the Airy beam and blue is the Gaussian beam. Since both beams can penetrate the mixture solution of 150 μm, the signal intensities of the two beams are not much different (the Airy beam has a slightly higher intensity than the Gaussian beam), but the Airy beam has better anti-scattering ability than the Gaussian beam, so the Airy beam has a better image resolution than the Gaussian beam (as shown in Figure 3c,e). The purple boxes in Figure 3c,e clearly show that the Airy beam has better image contrast than the Gaussian beam. The concave edge of the target number zero (purple box) is clearly identifiable in Figure 3c; however, it has become very fuzzy in Figure 3e. Figure 3h shows the relative intensity of the beam reflected through the 300 μm thick water/milk mixture over the target. Obviously, the signal strength of the Gaussian beam basically disappears, while that of the Airy beam does not significantly decrease and can still clearly image. The Airy beam has an obviously larger penetration depth and better anti-scattering ability than the Gaussian beam. Figure 3i shows the peak signals of the sample at different thickness water/milk mixture for Gaussian (blue triangles) and Airy (red circles) microscopy imaging. The decay rate of the Gaussian beam is faster than that of the Airy beam. The penetration depth of the Gaussian beam is approximately 135 μm and the Airy beam is 286 μm. The penetration depth of the Airy beam is approximately twice the penetration depth of the Gaussian beam.

**Figure 4:**
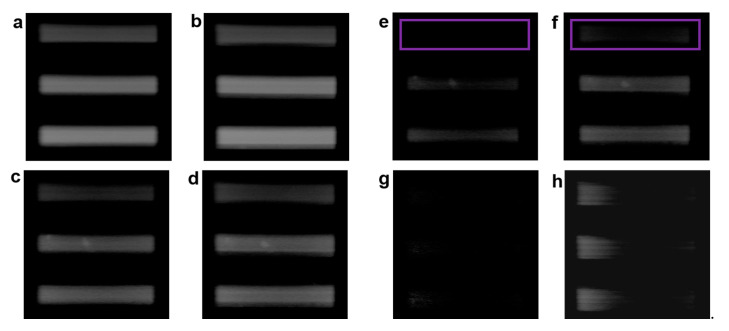
Relative intensity of the reflected light images from the target by line scanning with near-infrared laser (780 nm) Airy and Gaussian beams. (**a**) and (**b**) are images of the reflected light for the Gaussian and Airy beams without media, respectively; (**c**) and (**d**) are images for the Gaussian and Airy beam at 150 μm thick water/milk mixture, respectively; (**e**) and (**f**) are images for the Gaussian and Airy beam at 300 μm thick water/milk mixture, respectively; (**g**) and (**h**) are images for the Gaussian and Airy beam at 450 μm thick water/milk mixture, respectively.

Furthermore, we performed the experiments with near-infrared pulsed laser (780 nm, 80 MHz, 140 fs) for Airy and Gaussian modes. The experimental conditions remain the same as the 633 nm, but here we use a resolution plate (GCG-020101 Daheng Optics) as the sample. From Figure 4a,b, we can know that the two images of the Gaussian and Airy beams have no obvious difference. However, from the Figure 4c,d images, we also cannot see the obvious difference when the Gaussian and Airy beams through a 150 μm thick water/milk mixture, respectively. The reason is that the two beams both can penetrate the 150 μm mixture. From Figure 4e and Figure 3f, we can see that the two images have a significant difference when the Gaussian beam and Airy beam passing through a 300 μm thick water/milk mixture, respectively. The information of the image is partially missing (Purple box) in Gaussian mode, all information of the target also can be imaged in Airy mode, and the image of Airy mode has better image contrast and SNR than the image of Gaussian mode, which illustrates that the Gaussian beam almost reaches the penetration limitation at 300 μm. Figure 4g,h show that the image of Gaussian almost has no signal but the image of Airy has a part signal of the target when both beams through a 450 μm thick water/milk mixture, which illustrates that the Airy beam almost reaches the penetration limitation at 450 μm. Comparing with the result (270 μm) of Ref [24], we have a greater depth of transmission. In summary, the 780 nm Airy beam has a larger penetration depth and better anti-scattering ability than the 633 nm Airy beam in turbid media, and the Airy beam has a larger penetration depth and better anti-scattering ability than the Gaussian beam in turbid media.

**Figure 5:**
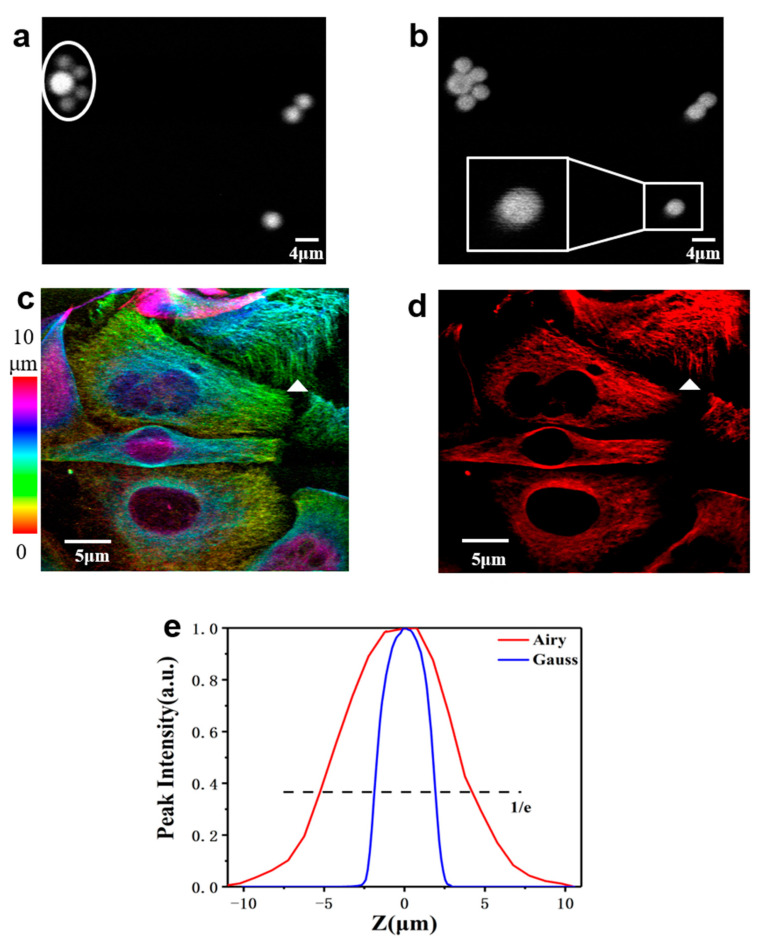
Volumetric imaging. (**a**) A single frame of fluorescent beads in the Gaussian mode. (**b**) Single frame of fluorescent beads in the Airy mode; (**c**) Projection of the Gaussian image stack of HeLa cell slice color-coded by depth; (**d**) Single frame of the HeLa cell in the Airy mode; (**e**) Peak signals of the fluorescent beads at different depths for the Airy beam (red curve) and the Gaussian beam (blue curve) microscopic imaging. Both signal intensities of the Airy beam and the Gaussian beam are normalized.

Finally, we performed one-photon (633 nm) fluorescence volumetric imaging with the Airy beam. We evaluated the system with 4 μm fluorescent beads (Tetraspeck Fluorescent Microspheres T14792 Invitrogen). Figure 5a,b are standard fluorescent bead images in the Gaussian and Airy mode, respectively. The fluorescent beads in the Airy mode clearly have a comet-like trailing shape (white box), and the large and the four small fluorescent beads differ in signal strength because they are not in a focal plane under the Gaussian mode (white circle, Figure 5a), whereas they are the same in the Airy mode because of the long focal length and the bent trajectory of the Airy beam [Figure 2d]. Then, the axial images of the fluorescent beads are stacked for mapping the lateral images. The transverse maximum signal intensities of the two trajectories along the propagation direction are shown in Figure 5e. The axial FWHMs of the Gaussian mode and Airy mode are 2.5 μm and 11.5 μm, respectively. The axial FWHM of the Airy beam is approximately 4 times larger than that of the Gaussian beam. To further show the advantage of the imaging with the Airy beam, we prepared HeLa cells as samples whose tubulin were labeled with Atto 647 N (Ex/Em: 647/664 nm). The home-built microscope was used to scan a thin layer of about 10 μm in a Gaussian mode with an axial step size of 0.5 μm. Figure 5c shows the 3D projection of the cell, the depth is coded in different colors for showing the 3D projection of the cells. A subset of these cells is recorded by a single frame using the Gaussian beam. Observing fast dynamic events in volume samples is still challenging, as it should take about 1 min to record such a 20-frame 3D stacked image. A single frame 2D image [Figure 5d] can image most of the cells in the same volume due to the elongated focus of Airy mode, and the microtubule structure of the HeLa cell is observable in the 3D image stack (white triangle). Such an imaging process only takes 1 s, which significantly increases the acquisition speed. Furthermore, the image position of the cell in the Airy mode has a tiny shift compared to the image under the Gaussian mode, which is caused by the bending axial trajectory of the Airy beam. More importantly, the images in both modes have a nearly identical lateral resolution (Although the lateral resolution with the Gaussian beam is a little better than that obtained with the Airy beam), and the Airy beam greatly expands the axial imaging range.

**Figure 6:**
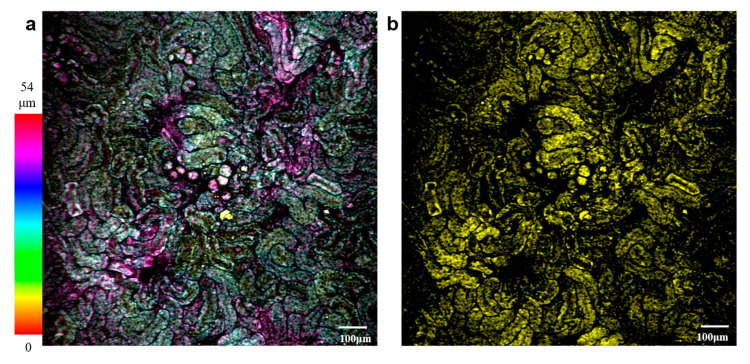
Two-photon volumetric imaging of mouse kidney tissue. (**a**) Projection of the Gaussian image stack of mouse kidney tissue color-coded by depth. (**b**) A single frame of the mouse kidney in the Airy mode.

To demonstrate the advantages of Airy beam volumetric imaging, we performed the dynamic two-photon fluorescence volumetric imaging of the isolated mouse kidney tissue with a 780 nm laser. The sample of the isolated mouse kidney tissue was soaked in Rhodamine 6G (Ex/Em: 518/543 nm, 1 mmol/L) for half an hour and rinsed by PBS for 3 times. Figure 6 is the two-photon volumetric imaging of mouse kidney tissue, Figure 6a shows the projection of the Gaussian images stack (27 frames) of the mouse kidney tissue, the imaging depth is about 54 μm and is coded in different colors for showing the 3D projection of the tissue. Figure 6b is the 2D volumetric image of the mouse kidney tissue with an Airy beam. From Figure 6b, we can see that the information of the volumetric image almost covers Figure 6a. Therefore, a single 2D volumetric image of Airy mode can be used to replace the projection image of the 3D image with 27 frames Gaussian images. Furthermore, we use the two-photon Airy beam to monitor the dynamic changing of the mouse kidney tissue (See Video S1). According to the two-photon volumetric imaging experiment, we can obviously know that the two-photon volumetric imaging of the Airy beam can significantly increase the acquisition speed and avoid the side effect of motion blur in in vivo imaging. It will have a broad application prospect in the dynamic imaging of live tissues.

## 4. Conclusions

In conclusion, we have demonstrated microscopic imaging with the Airy beam, which expands the axial working range when there is no significant difference in lateral resolutions. Compared to the Gaussian beam, the non-diffracting Airy beam has a larger penetration depth and better anti-scattering ability while imaging strong scattering samples. Because the Airy beam has a long focal length, the volumetric imaging range in the axial direction is enough long to cover more structures in only one frame. The elongated focal length provides a greater depth of view, which greatly increases the volumetric imaging rate. Moreover, the fluorescence microscopic imaging with the Airy beam can capture all axial images in the effective focal length range, so it is more robust for live sample imaging, avoiding axial motion of samples. Overall, these advantages make laser scanning microscopy with non-diffracting Airy beams as a potential tool to monitor the deep biological activities in real-time in a large volume.

## Data Availability

The raw data supporting the conclusions of this article will be made available by the authors, without undue reservation.

## Supplementary Materials

The following are available online at https://www.mdpi.com/article/10.3390/membranes11060391/s1, Video S1. Dynamic two-photon volumetric imaging of mouse kidney tissue with Airy beams.
